# Acute nicotine administration effects on fractional anisotropy of cerebral white matter and associated attention performance

**DOI:** 10.3389/fphar.2013.00117

**Published:** 2013-09-18

**Authors:** Peter Kochunov, Xiaoming Du, Lauren V. Moran, Hemalatha Sampath, S. Andrea Wijtenburg, Yihong Yang, Laura M. Rowland, Elliot A. Stein, L. Elliot Hong

**Affiliations:** ^1^Department of Psychiatry, Maryland Psychiatric Research Center, University of Maryland School of MedicineBaltimore, MD, USA; ^2^Department of Physics, University of MarylandBaltimore County, MD, USA; ^3^National Institute on Drug Abuse, Neuroimaging Research BranchBaltimore, MD, USA

**Keywords:** nicotine, DTI-FA, white matter, acute change, cognition, processing speed, attention

## Abstract

**Introduction**: Nicotinic acetylcholine receptors are present in the cerebral white matter (WM). We hypothesized that WM response to nicotine can be detected by diffusion tensor imaging (DTI); and that such responses may be associated with nicotine-led cognitive enhancement in sustained attention.

**Methods**: A randomized, nicotine-placebo patch, crossover, double-blind clinical trial in two non-overlapping cohorts of smokers was used to test the hypothesis. The discovery cohort consisted of 39 subjects (*N* = 20/19 controls/schizophrenic patients, age = 36.8 ± 10.1 years) and the replication cohorts consisted of 38 healthy smokers (31.7 ± 10.5 years). WM integrity was measured by fractional anisotropy (FA) values for the whole brain and nine preselected WM tracts using tract-based-spatial-statistics.

**Results**: Nicotine significantly enhanced FA values for the genu of corpus callosum compared with placebo (ΔFA_genu_) (*p* = 0.01) in smokers with low recent smoking exposure as measured by low average cotinine level. This finding was replicated in the second cohort (*p* = 0.02). ΔFA_genu_ values explained 22% of variance in performance of a sustained attention task during the nicotine session (*p* = 0.006). However, this effect was limited to schizophrenia patients (*r* = 0.62 and 0.09; *p* = 0.003 and 0.7 for patients and controls, respectively).

**Conclusion**: Acute pharmacological influence of nicotine patch on WM integrity appeared present, but was dependent on nicotine intake from recent smoking. Change in the WM integrity in the genu of corpus callosum was associated with a significant proportion of variability of nicotine-led changes in sustained attention/working memory of the smokers. Further studies will be necessary to understand biophysical underpinning of the nicotine-related changes in FA.

## Introduction

Pharmacological effects of nicotine on the central nervous system are traditionally measured by its impact on synaptic terminal nicotinic acetylcholine receptors (nAChRs) located within gray matter structures. However, PET ligand and histological binding studies have demonstrated that nAChRs are present in the cerebral white matter (WM) in both human and non-human primates (Ding et al., 2004; Pimlott et al., 2004; Kimes et al., 2008; Hillmer et al., 2011). Non-synaptic nAChRs have been thoroughly studied in the peripheral (Lang et al., 2003) and central (Zhang et al., 2011a) nervous systems. Nicotinic agonist applied to the axonal trunks led to increased axonal excitability, axonal Ca^2+^ influx (Zhang et al., 2004), and modulation of action potentials, suggesting these receptors are functional (Vizi and Lendvai, 1999; Zhang et al., 2004). Axonal application of nAChR antagonist blocks this effect (Vizi and Lendvai, 1999), indicating a direct axonal nAChR functional effect. Here, we employed a standard clinical trial design to evaluate two hypotheses in humans. First, we hypothesized that an acute pharmacological effect of nicotine may be observed in cerebral WM using diffusion tensor imaging (DTI). Second, we hypothesized that the enhancing effects of nicotine on cognition may potentially be associated with nicotine-related changes in the cerebral WM. Nicotine administration is known to enhance cognition, with improved sustained attention being one of the most consistent findings, especially in patients with schizophrenia (Sacco et al., 2005; Hong et al., 2011). Our analysis included a cohort of patients with schizophrenia because patients affected with this disorder show both larger sustained attention deficits and nicotine-related cognitive enhancement than normal controls (Levin et al., 1996; Hong et al., 2011).

Acute nicotine effects on cerebral WM have previously never been reported, although substantial literature is available on examining the effect of chronic smoking and WM changes. Neuroimaging research in smokers has linked smoking to changes in Fractional Anisotropy (FA) of water diffusion, which is a sensitive index of WM integrity (Basser, 1994; Ulug et al., 1995; Conturo et al., 1996; Pierpaoli and Basser, 1996; Kochunov et al., 2007). Seemingly contradictory effects of smoking on FA have been observed. Most studies in chronic and heavy smokers reported reduced FA values when compared to non-smokers (Swan and Lessov-Schlaggar, 2007; Hudkins et al., 2010; Kim et al., 2010; Cullen et al., 2011; Gons et al., 2011; Liao et al., 2011; Zhang et al., 2011a,b). However, light smoking and smoking in adolescents have been associated with FA increases when compared to age and gender matched non-smokers (Paul et al., 2008; Hudkins et al., 2010; Liao et al., 2011). We propose that it is possible that shorter exposure to nicotine in the adolescents (Jacobsen et al., 2007; Cullen et al., 2011) or lighter smoking (Paul et al., 2008) was associated with increased WM-FA values possibly because nicotine may have a net effect on enhancing FA; while in more chronic or heavier smokers, nicotine-related FA change could probably be negated by the multitude of harmful effects of chronic smoking.

## Methods

### Discovery cohort

#### Participants

DTI data were collected in 39 adult smokers, including 20 normal controls (17/3 M/F, age = 36.5 ± 10.5 years) and 19 age and sex frequency-matched schizophrenia patients (17/2 M/F, age = 37.2 ± 10.9 years) (Table 1), during a clinical trial that studied nicotine's effect on cognitive functions under fMRI (Hong et al., 2011). All subjects were habitual smokers, consuming 19.5 ± 8.2 cigarettes per day (CPD) and with a history of 19.8 ± 16.6 pack/years (Table 1). Normal controls were recruited through media advertisements. There were no significant patients vs. controls differences on any of smoking related measurements (two-tailed *t*-test *p* > 0.5). Patients were recruited from local mental health outpatient clinics. All participants were evaluated by the Structured Clinical Interview for DSM-IV. Subjects with current alcohol and/or substance dependence or abuse were excluded. Schizophrenia patients were clinically stable, medicated on antipsychotic medication, and had no change in medications or dosage of their medications between the two DTI sessions (Table 1). Normal controls had no DSM-IV Axis I diagnosis. All subjects signed written informed consent approved by local IRB panels.

**Table 1:** **Subjects' demographic and clinical information including gender, age, cotinine level, current antipsychotic medication dose, as calculated by chlorpromazine equivalent (CPZ), and Fagerstrom Test for Nicotine Dependence (FTND) scores, a measure of smoking addiction severity**.

	**Discovery cohort**				**Replication cohort**			
	**Whole sample**	**Low cotinine**	**High cotinine**	***p*-value**	**Whole sample**	**Low cotinine**	**High cotinine**	***p*-value**
Subjects (M/F)	39 (34/5)	17 (15/2)^*^	17 (14/3)^*^	0.6	38 (18/20)	19 (10/9)	19 (8/11)	0.5
Cotinine (±sd)	321.7 ± 143.1	208.1 ± 77.4	428.4 ± 94.4	1.00E-08	283.2 ± 102.0	194.9 ± 94.1	363.6 ± 73.5	2.00E-07
Age (±sd)	37.1 ± 10.6	39.1 ± 10.8	35.2 ± 10.4	0.32	31.8 ± 7.6	31.6 ± 7.3	31.9 ± 8.1	0.9
Patients/controls	19/20	7/10^*^	10/7^*^	0.4	N/A	N/A	N/A	N/A
CPZ (±sd)	397.4 ± 237.8	427.4 ± 263.8	379.2 ± 233.6	0.7	N/A	N/A	N/A	N/A
FTND (±sd)	4.6 ± 2.1	3.8 ± 2.2	5.4 ± 1.7	0.005	5.9 ± 2.3	5.1 ± 1.9	6.8 ± 2.3	0.02
Pack years (±sd)	19.8 ± 16.6	19.0 ± 15.1	21.2 ± 18.4	0.7	15.2 ± 9.7	13.9 ± 7.6	16.6 ± 11.6	0.4

*The two cohorts were further subdivided into high and low average cotinine groups. P-values were from two-tailed t-test or χ^2^ test, to evaluate difference between high and low cotinine subgroups*.

* *Average cotinine measurements were available for 34 subjects in the first sample, including 17 patients and 17 controls*.

#### Study design

**Figure 1:**
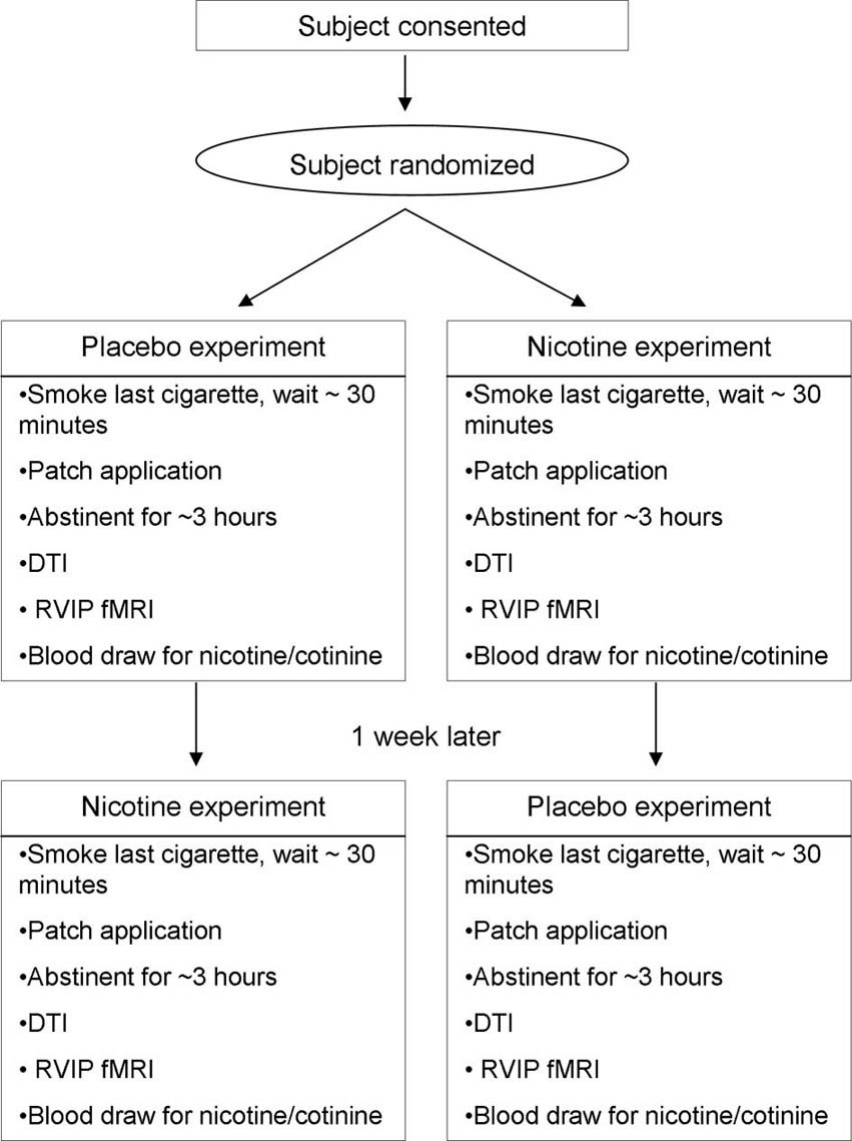
**Schematic diagram of the clinical trial procedure for the discovery sample.** The procedure for the replication sample was essentially the same, but without the RVIP fMRI.

DTI data were evaluated in two sessions during placebo and nicotine patch application in a double-blind, randomized, cross-over design, about 1 week part (Figure 1). Subjects maintained their usual smoking pattern on each scanning session until about 1 h prior to the administration of transdermal patch and then wore the patch and abstained from further smoking for 4.5 h (2.5 h before + 2 h during imaging). Carbon monoxide (CO) levels were measured immediately prior to patch applications. Subjects who smoked greater than 15 CPD received 35 mg of nicotine while those smoking between 10 and 14 CPD received a patch with 21 mg of nicotine (Nicoderm CQ, GlaxoSmithKline, NC), an approach we used to better approximate the nicotine dose to smoking severity. The appearance of the placebo patches was matched to that of the nicotine patches. After patch application, the subject waited for 2.5 h to achieve steady serum level of nicotine. The subject was then positioned in the scanner and underwent an 8-minute T1 scan and an 8-minute DTI scan, followed by other behavioral fMRI scans that are described elsewhere (Hong et al., 2011). DTI data was therefore collected after about 3 h of nicotine patch or about 4 h from last cigarette smoking.

Immediately following the completion of an imaging session, venous blood was drawn to measure blood-levels of nicotine and cotinine. Nicotine exposure from two sources was quantified: from the acute patch vs. from chronic cigarette smoking (Hong et al., 2011; Schnoll et al., 2011). The individual nicotine absorption from the nicotine patch was quantified by subtracting nicotine levels during the nicotine session from that during the placebo session. The individual nicotine intake from recent smoking was estimated by averaging the cotinine levels taken from the 2 sessions. This is because nicotine has a short half-life (~60 min) and therefore its serum level is a sensitive measurement of short-term nicotine exposure. Cotinine level was used to quantify recent nicotine exposure from smoking (Schnoll et al., 2011). Cotinine has a long half-life (~20 h) and its levels will therefore be more sensitive to recent cumulative nicotine intake from smoking rather than from patch absorption. Nicotine measurements for six subjects and cotinine measurements for five subjects were not available due to various technical problems. To monitor side effects and withdrawal symptoms, a self-reported symptom checklist and a self-reported mood change questionnaire (Parrott et al., 1996) were administered before patch application and after scan.

#### Imaging

Imaging data was collected using a Siemens 3T Allegra MRI (Erlangen, Germany) located at the National Institute on Drug Abuse (NIDA), NIH using a quadrature RF head coil. DTI data was collected using a spin-echo, EPI sequence with a spatial resolution of 1.7 × 1.7 × 4.0 mm. The sequence parameters were: *TE*/*TR* = 87/5000 ms, *FOV* = 200 mm, axial slice orientation with 35 slices and no gaps, twelve isotropically distributed diffusion weighted directions, two diffusion weighting values (*b* = 0 and 1000 s/mm^2^) and one *b* = 0 image. Five set of images were collected for the subsequent averaging to improve signal to noise. Subjects' head movement was minimized by the use of foam padding, a vacuum bag, bite bar, and/or hardened polyurethane foam.

#### Processing of DTI data

A tract-based spatial statistics (TBSS) method, distributed as a part of FMRIB Software Library (FSL) package, was used for tract-based analysis of diffusion anisotropy (Smith et al., 2006). First, FA images were created by fitting the diffusion tensor to the raw diffusion data. In the next step, all FA images were globally spatially normalized to the John Hopkins University (JHU) that is distributed with the FSL package (Wakana et al., 2004) and then non-linearly aligned to a group-wise, minimal-deformation target (MDT) brain as detailed elsewhere (Jahanshad et al., 2013). The global spatial normalized was performed using a method distributed with FSL package (FLIRT) (Smith et al., 2006) with 12° of freedom. This step was performed to reduce the global intersubject variability in brain volumes prior to non-linear alignment. The group's MDT brain is identified by warping all individual brain images in the group to each (Kochunov et al., 2001). The MDT is selected as the image that minimizes the amount of the required deformation from other images in the group. Next, individual FA images are averaged to produce a group-average anisotropy image. This image is used to create a group-wise skeleton of WM tracts. The skeletonization procedure is a morphological operation, which extracts the medial axis of an object. This procedure is used to encode the medial trajectory of the WM fiber-tracts with one-voxel thin sheaths.

Finally, FA images were thresholded at *FA* = 0.20 level to eliminate non-WM voxels and FA values were projected onto the group-wise skeleton of WM structures. This step accounts for residual misalignment among individual WM tracts. FA values are assigned to each point along a skeleton using the peak value found within a designated range perpendicular to the skeleton. The FA values vary rapidly perpendicular to the tract direction but very slowly along the tract direction. By assigning the peak value to the skeleton, this procedure effectively maps the center of individual WM tracts on the skeleton. This processing is performed under two constraints. A distance map is used to establish search borders for individual tracts. The borders are created by equally dividing the distance between two nearby tracts. Secondly, a multiplicative 20 mm full width at half-max Gaussian weighting is applied during the search to limit maximum projection distance from the skeleton. Beside FA, we also explored radial and axial diffusivity.

#### Estimation of intra-session motion

Average head motion during the DTI scans were measured by spatial alignment of diffusion-sensitized (*b* = 1000) images to the *b* = 0 image. Automated, nine-parameter (three rotations, three scales, and three translations) FLIRT spatial normalization method was used with default parameters. Next, RMSDIFF (Smith et al., 2004) was used to estimate the root mean square (RMS) movement distance between diffusion sensitized and *b* = 0 images. The RMSDIFF program calculates the RMS difference in transformed locations using a simple spherical brain model that spanned 25% of the space. The 4 × 4 alignment matrices for diffusion-sensitized images were compared to a 4 × 4 identity matrix, which served as the no-movement reference. The RMS distance calculation accounted both translation and rotation providing a sensitive index of head motion.

#### Tract-based analysis

**Figure 2:**
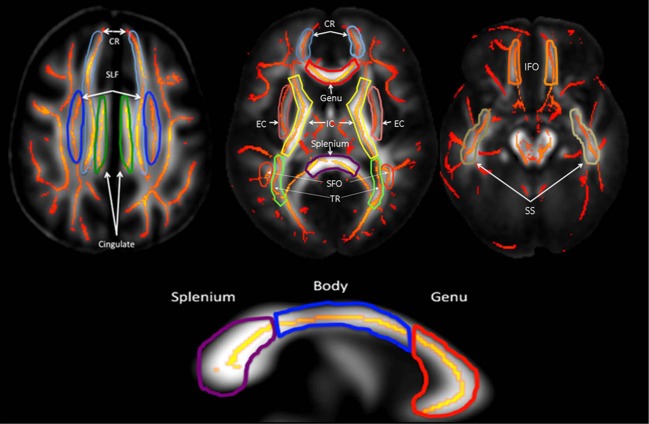
**Average DTI FA values were measured for ten major cerebral WM tracts (Table 1)**.

To explore tract-specific effect, the population-based, 3D, DTI cerebral WM tract atlas developed in John Hopkins University (JHU) and distributed with the FSL package (Wakana et al., 2004) was used to calculate population average FA values along the spatial course of nine, largest, core WM tracts (Table 2, Figure 2) as described elsewhere(Kochunov et al., 2010, 2011a). These tracts were chosen based on their size (>10 cm^3^) and their presence in all subjects, which simplifies multi-subject analysis (Wakana et al., 2004; Kochunov et al., 2011b). The details of the by-tract measurements are detailed elsewhere (Jahanshad et al., 2013). Per-tract average values were calculated by averaging the values along the tracts in both hemispheres as detailed in our prior work (Kochunov et al., 2011b; Jahanshad et al., 2013). The overall average FA values were calculated by averaging values for the entire WM skeleton.

**Table 2:** **White matter tracts used in the analysis**.

**Tract**	**Fiber type**	**Connections**
Genu, body, and splenium of corpus callosum	C	Cerebral hemispheres
Cingulum (Cing)	A	Cingulate gyrus/Hippocampus
Corona radiata (CR)	P	Cortical/Subcortical
External capsule (EC)	A	Frontal/Temporal/Occipital
Internal Capsule (including thalamic radiation) (IC)	P	Subcortical/Brainstem/Cortex
Superior/Inferior fronto-occipital fasciculi (SFO and IFO)	A	Frontal/Parietal/Occipital
Superior longitudinal fasciculus (SLF)	A	Frontal/Temporal/Occipital

*C, Commissural; P, Projection; A, Association*.

#### Sustained attention

After DTI and T1 MPRAGE acquisition, a rapid visual information processing task (RVIP) was administrated to examine the effect of nicotine on sustained attention. The RVIP is a continuous performance task (CPT) with a predominantly sustained attention component and working memory load (Wesnes and Warburton, 1983). Briefly, we showed (Hong et al., 2011) that administration of nicotine enhanced accuracy (*p* = 0.001) and processing speed (*p* = 0.03) compared to placebo in schizophrenia patients and also enhanced accuracy (*p* = 0.05) but not processing speed (*p* = 0.11) compared with placebo in healthy controls, but there was no significant treatment × diagnosis interaction.

#### Statistical analysis

Significance of treatment effect (nicotine vs. placebo) was calculated using a paired *t*-test. Intersession differences in FA values were calculated as the percent change over the average for both sessions. Significance of the predictors on the intersession differences in FA values was assessed using linear correlation analysis. The above was calculated for the whole brain and for the tract-specific analyses. Finally, effects of nicotine administration on cerebral WM integrity were studied using a repeated measure ANOVA where FA values in the nicotine and placebo conditions were treated the repeated measures. Significant values were corrected for ten comparisons for whole brain + nine tracts (*p* = 0.05/10 = 0.005).

#### Replication cohort

*S*ignificant FA findings from the discovery sample were tested in a separate, non-overlapping cohort of 38 healthy smokers (18/20 M/F, age = 31.7 ± 10.5 years) (Table 1). All subjects were habitual smokers, consuming 20.9 ± 6.6 CPD, which was not statistically different from the discovery cohorts (*p* = 0.7). Subjects for the replication cohort were recruited through media advertisements. The study design closely matched that in the discovery cohort; however, a 21 mg nicotine patch was used in all subjects. The DTI data were collected on the same scanner and the head coil but using a different version DTI sequence. The same sequence control parameters were used with exception that one fewer (4 vs. 5) set of images was collected for averaging. DTI data processing was the same as in the replication cohort. Statistical analyses in this cohort were aimed at replication of the significant findings identified in the discovery cohort.

## Results

### Discovery cohort

No diagnosis-by-treatment interactions were found in imaging or nicotine related measures (all *p* > 0.20), therefore, schizophrenia patients and controls were combined. There was an 8-fold increase in serum nicotine concentration in the nicotine vs. placebo sessions (35.2 ± 11.6 vs. 4.1 ± 4.4 ng/ml, respectively; paired *t*-test *p* < 1 × 10^−10^). The serum cotinine levels were 35% higher during the nicotine than during the placebo sessions (361.2 ± 141.6 vs. 267.9 ± 135.9 units/ml, respectively; *p* = 0.01). Cotinine levels for two visits were highly correlated (*r* = 0.75; *p* < 1 × 10^−7^) despite the 8-fold difference in nicotine levels, suggesting that this measurement primarily quantifies recent cumulative nicotine exposure from smoking rather than acute nicotine from the patch. Therefore, the cotinine levels were averaged to produce a single index of recent cumulative nicotine exposure from smoking. Whole-brain average diffusion parameters demonstrated excellent (*r*^2^ > 0.9) intersession reproducibility. The highest reproducibility was observed for the axial diffusivity (*r*^2^ = 0.95) and the lowest was seen for the FA values (*r*^2^ = 0.92). Intersession reproducibility for the FA values in the genu of corpus callosum were likewise high for both the discovery and replication cohorts (*r*^2^ = 0.93, 0.90 and.85 for axial and radial diffusivities and FA, respectively).

**Figure 3:**
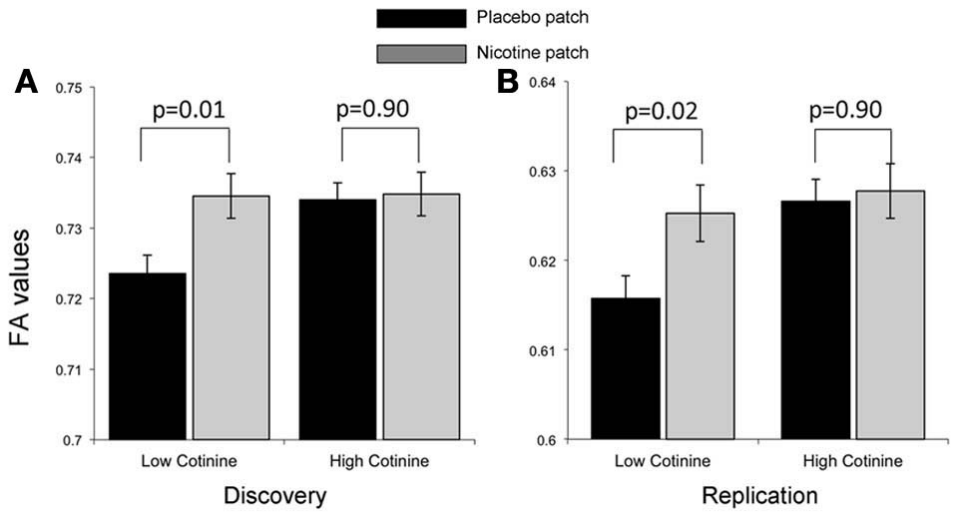
**Treatment effect on FA values for the genu of corpus callosum by comparing placebo (black bar) and nicotine (gray bars) showed a significant interaction with average cotinine level in the discovery cohort (A).** Subjects in the discovery cohort were separated into two (low and high cotinine) equal groups by sorting the data based on cotinine levels, with the average cotinine levels = 208 ± 77 and 428 ± 94 mg/ml, respectively. Nicotine enhanced the FA in the low-cotinine group (*p* = 0.01) but not in the high-cotinine group. **(B)** The low and high cotinine groups in the replication cohort had average cotinine levels = 194 ± 49 and 364 ± 73 mg/ml, respectively. Note that the discovery and replication cohorts were collected under different DTI protocols, leading to a systematic difference in the average FA values.

Correlation between the average cotinine level and ΔFA (nicotine vs. placebo) in the whole brain and the nine WM tracts showed that FA change at the genu of corpus callosum (ΔFA_genu_) from placebo to nicotine patch was significantly correlated with average cotinine level (*p* < 0.001; Table 3), which was the only significant finding after corrected for *N* = 10 comparisons. None of the correlation between ΔNicotine and ΔFA in the whole brain or the nine WM tracts was significant after correction of multiple comparisons. A repeated measure ANOVA using genu FA in the nicotine and placebo conditions as the repeated measures and the average cotinine level was the covariate (Figure 3, left) reported that both the main effect of treatment and treatment × cotinine interaction were significant (*F* = 8.9, *p* = 0.005 and *F* = 11.6, *p* = 0.002, respectively; Greenhouse–Geisser corrected). To examine the significant interaction, we divided subjects into high-and-low cotinine groups, consisting of 17 subjects each, using median split of the averaged cotinine (Table 1, Figure 3). Using median split of cotinine levels for either of the visits resulted in the same group constitution. Nicotine enhanced the FA at the genu in subjects in the low-cotinine group (two tailed, paired *t*-test, *p* = 0.01)but not in the high-cotinine group (two tailed, paired *t*-test *p* ≈ 0.9). Therefore, nicotine enhanced FA only when the smokers' baseline level of cotinine was low; and this effect was not present when cotinine level was high.

**Table 3:** **Correlation coefficients for the Δwhole-brain and Δtract-wise FA value and Δnicotine and average cotinine levels for the discovery cohort**.

**FA**	**ΔWhole Brain**	**ΔGenu**	**ΔBody**	**ΔSplenium**	**ΔCing**	**ΔCR**	**ΔEC**	**ΔIC**	**ΔSFO and IFO**	**ΔSLF**
ΔNicotine	−0.29 (0.10)	0.28 (0.12)	0.16 (0.4)	0.26 (0.14)	−0.42 (0.01)^*^	−0.36 (0.04)^*^	21 (0.2)	−0.23 (0.16)	−0.21 (0.2)	−0.24 (0.2)
(*N* = 33)										
Average cotinine	−0.12 (0.5)	−0.59 (0.0001)^**^	0.01 (0.9)	0.18 (0.3)	−0.12 (0.5)	0.12 (0.5)	−0.01 (0.9)	−0.02 (0.8)	0.02 (0.9)	0.04 (0.8)
(*N* = 34)										

* *Nominally significant*.

** *Significant after correction for N = 10 comparisons*.

Change in self-reported withdrawal symptoms was not significantly different between sessions (6.9 ± 0.7 vs. 7.1 ± 0.8, paired *t*-test *p*-value = 0.25 for nicotine and placebo sessions, respectively) and were not significantly correlated with whole-brain or by-tract ΔFA (all *p* > 0.3). Patients and controls showed no difference in the severity of withdrawal symptoms (*p* = 0.5). Systolic blood pressure (BP) was elevated from the placebo to the nicotine session (126 ± 13.6 vs. 135.6 ± 13.8 mmHg, respectively; *p* = 0.005); but not for diastolic BP (*p* = 0.11). There were no significant correlations between ΔFA and ΔBP (*p* > 0.2). The subjects' motion averaged 0.66 mm during nicotine and 0.65 mm during placebo sessions (paired *t*-test *p* = 0.60) and the expected effects of this motion on FA values are < 0.1% (Muller et al., 2011). Therefore, there were no significant differences in head motion between sessions or more importantly, head motion contribution to the ΔFA findings.

### Replication cohort

There was a 7-fold increase in serum nicotine concentration in the nicotine vs. placebo sessions (33.6 ± 8.4 vs. 5.0 ± 4.1 ng/ml, respectively; paired *t*-test *p* < 1 × 10^−10^). The serum cotinine levels were 33% higher during the nicotine than during the placebo sessions (320.2 ± 114.9 vs. 241.2 ± 94.3 units/ml, units/ml, respectively; *p* = 1 × 10^−4^). Cotinine levels measured at the placebo and nicotine were as highly correlated (*r* = 0.78, *p* < 1 × 10^−7^) as in the discovery cohort (*r* = 0.75). The nicotine-related parameters were similar to that of the discovery cohort with no significant differences in the Δnicotine and average cotinine levels between two cohorts (Δnicotine: 31.6 ± 11.7 vs. 29.8 ± 8.34 ng/ml; average cotinine: 321.7 ± 143.1 vs. 283.2 ± 102.1 units/ml, for discovery and replication cohorts, respectively, all *p* > 0.2). The correlation between average cotinine levels and ΔFA_genu_ was again significant (*r* = −0.39, *p* = 0.02). Significant main effects of treatment and treatment × cotinine interaction were also observed in the replication cohort (*F* = 9.1, *p* = 0.005 and *F* = 6.2, *p* = 0.02). The ΔFA_genu_ values in smokers with low-cotinine level was significantly higher during nicotine then in placebo sessions (two tailed, paired *t*-test, *p* = 0.02; Table 1, Figure 3) and this effect was absent in smokers with high average cotinine level (two tailed, paired *t*-test *p* ≈ 0.9).

### FA change and sustained attention

**Figure 4:**
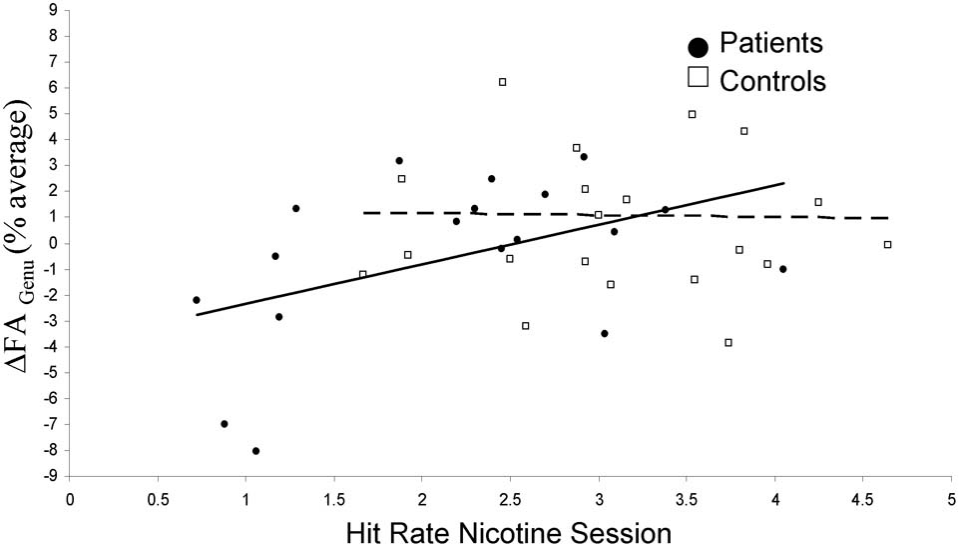
**Nicotine-related changes in FA values of the genu of corpus callosum (ΔFA_Genu_) are plotted against the hit rate during nicotine session, separately in schizophrenia patients (solid point/line) and normal controls (hollow points/dashed line)**.

The hypothesis that nicotine-led change in WM integrity is associated with enhancement in cognition was tested by correlating ΔFA_genu_ with RVIP hit rate and reaction time. Both measures are enhanced by nicotine (Hong et al., 2011). The hit rate in the nicotine condition was significantly correlated with ΔFA_genu_ (*r* = 0.44, *p* = 0.005) (Table 4), significant after Bonferroni correction for *N* = 6 comparisons. Additionally, ΔFA_genu_ was correlated with the intersession difference in the reaction time in the controls (*r* = 0.32, *p* = 0.03), although this was not significant after Bonferroni correction. In both instances, increases in WM integrity during nicotine session corresponded to enhanced performance. A *post-hoc* analysis showed that the relationship between ΔFA_genu_ and hit rate was more pronounced in the schizophrenia patients (*r* = 0.62; *p* = 0.003) than in the normal controls (*r* = 0.09; *p* = 0.7) (Figure 4). We evaluated if either placebo or nicotine session measures of withdrawal symptoms, systolic and diastolic BP and motion or their intersession difference could be associated with cognitive enhancement but observed no significant correlations between them and RVIP hit rate and reaction time, or their intersession differences (all *r* < 0.10, all *p* > 0.5). Additionally, there were no significant relationships between the dose of antipsychotic medication and neurobehavioral performance measurements (all *r*-values <0.15; all *p* > 0.4) (Hong et al., 2011). Nicotine related changes in ΔFA_genu_ were mainly driven by changes in radial diffusivity, which also demonstrated significant correlation with the hit rate in the nicotine condition (*r* = 0.45, *p* = 0.004).

**Table 4:** **Correlation coefficients for intersession difference in FA values for the genu and corpus callosum and measurements of sustained attention and reaction time**.

	**Hit rate nicotine patch**	**Hit rate placebo patch**	**ΔHit rate**	**Reaction time nicotine patch**	**Reaction time placebo patch**	**ΔReaction time**
ΔFA_Genu_ (*N* = 38)	0.44 (0.006)^**^	0.29 (0.08)	0.17 (0.30)	−0.24 (0.15)	−0.22 (0.20)	0.35 (0.03)^*^

Δ: Difference between nicotine and placebo patch sessions

* *Nominally significant*.

** *Only the correlation with hit rate during the nicotine patch session was considered statistically significant after correction for six comparisons*.

## Discussion

Our study demonstrated that a subtle change in FA values for the genu of corpus callosum (ΔFA_genu_) between nicotine and placebo patch sessions had unexpectedly interacted with the average cotinine levels, a measure of recent nicotine intake and its metabolism from cigarette smoking (Schnoll et al., 2011). Subjects with lower serum cotinine level experienced a significant increase in FA_genu_ values due to administration of a single dose of nicotine when compared with placebo and subjects with high serum cotinine level experienced no change in FA_genu_ values. The interpretation of this finding is not straightforward without the knowledge of the underlying pharmacological mechanisms but it was replicable in an independent sample and was not associated with head motion, blood pressure, and/or withdrawal symptoms experienced by the subjects. Further, the ΔFA_genu_ was significantly and positively correlated with sustained attention performance during nicotine patch administration but this finding was observed only in schizophrenia patients. To our knowledge, this is the first study to demonstrate and replicate acute pharmacological effects of nicotine on cerebral WM. This study is also the first demonstration that an acute pharmacological effect on WM integrity can be associated with changes in cognitive performance.

The biophysical mechanisms behind the observed effects are unknown. The absolute FA values are sensitive to many parameters including myelin content, intra-voxel axonal crossing and axonal fiber density and diameter (Beaulieu, 2002). However, recent work from our group, based on theoretical modeling of diffusion properties by Sukstanskii et al. (2003, 2004), led to a development of a permeability-diffusity model of diffusion signal (Kochunov et al., 2013). There, we showed that the permeability-diffusivity index, a measurement that is theoretically sensitive to permeability of cellular membrane, yielded more robust patient-control differences in schizophrenia than FA values (Kochunov et al., 2013). Moreover, this work showed that FA values were significantly correlated with PDI suggesting that FA may in part be sensitive to the activity of axonal ion-channels. This is consistent with observations made in animal models of ischemic stroke. Under normal physiological condition, the axonal membranes are semi-permeable by the active exchange via ionic and water pumps (Baslow, 2002). Ischemia leads to failure of molecular pumps and restriction of active permeability. This failure of molecular pumps is observed as a rapid (within minutes) drop in both radial and axial diffusivity and the eventual drop in FA (20–50%) (Li et al., 2000). Here, we speculate that nicotine-related changes in FA values may be driven by changes in active axonal permeability due to pharmacological stimulation of nAChRs located within cerebral WM (Anderson et al., 1996; Mandl et al., 2008). For example, nAChR α 5, α 3, and β 4 are expressed in unmyelinated human C fibers (Benarroch and Low, 1991; Lang et al., 2003); and α 4β 2 and α 7 are expressed in optical nerve axons. Human PET (Ding et al., 2004) and postmortem human brain ligand binding studies show unequivocal WM presence of nAChRs (Pimlott et al., 2004).

This potential mechanism of nicotine-related FA change due to changes in axonal permeability is also supported by the evidence that axonal nAChRs regulate calcium influx (Edwards and Cline, 1999). Nicotine applied directly to rodent optic nerve axons increases intra-axonal calcium (Zhang et al., 2004). The axonal nAChR induced calcium transients have a sustained effect in modulating action potentials passing through the axons (Zhang et al., 2004), providing a plausible mechanism to modulate cognitive performance. Alternatively, nicotine effect on FA may also be due to nicotine stimulation of the nAChRs located at the synaptic terminals. Finally, nAChRs are also widely expressed in oligodendrocytes and astroglial cells of cerebral WM (Hassel et al., 2008). Stimulation of glial nAChR inhibits glycogen synthase kinase 3 (GSK3) and activates Akt/GSK3 myelination pathway (Bartzokis, 2012), (Flores et al., 2008; Narayanan et al., 2009) and this may also contribute the observed FA effect. These or other, yet unidentified, mechanisms may also contribute to the correlation between FA change and cognition. The exact mechanism cannot be ascertained here, but this finding should encourage future research to examine how changes in WM integrity may or may not contribute to the overall nicotine effects on brain and cognition.

Nicotine is known to improve sustained attention (Levin et al., 1996; Dépatie et al., 2002; Smith et al., 2002; Harris et al., 2004; Sacco et al., 2005; Barr et al., 2008). Previous study from our group demonstrated that nicotine improved accuracy and reaction time, especially in schizophrenia patients (Hong et al., 2011). Using the DTI approach, the nicotine effect on genu FA is now observed to correlate with sustained attention performance, explaining 22% of the variance in hit rate during the nicotine administration. It is important to emphasize that the relationship should not be interpreted as causative, predictive, or modulatory. It is fundamentally a correlational finding that may or may not be causally related. This said, the pattern of correlation suggests that subjects with a higher ΔFA_genu_ show a proportionally higher sustained attention in schizophrenia patients. There ΔFA_genu_ explained 40% of the sustained attention variance during nicotine administration. In normal controls this trend was positive but not significant. The observation that the robust effect of the change in FA of genu on processing is meaningful because the genu makes up the majority of fibers for pre-frontal and frontal inter-hemispheric signal traffic (Aboitiz et al., 1992). These commissural fibers facilitate inter-hemispheric connectivity and are thought to modulate the speed of processing of cognitive information (Pfefferbaum et al., 2000; Kochunov et al., 2009; Salthouse, 2009). The nominally significant correlation between ΔFA_genu_ and reaction time (ΔRT) (*p* = 0.03) may support this notion. It is possible that the micro-architectural nature of the genu, such as the presence of densely packed, thinly myelinated fibers (Aboitiz et al., 1992), is what leads to large FA changes in response to the acute pharmacological effects of nicotine.

The finding of significantly higher effect of ΔFA_genu_ on cognition in schizophrenia patients is consistent with the findings that administration of nicotine in patients significantly improved (*p* < 0.001) their performance, while the nicotine-related change in normal controls was only nominally significant (*p* = 0.05) (Hong et al., 2011). Additionally, schizophrenia patients showed a significantly lower (*p* < 0.0001) performance on the sustained attention task during placebo session thereby setting a lower baseline (Hong et al., 2011). We did not observe a significant correlation between ΔFA_genu_ and the hit rate during the placebo condition, however, this correlation was nominally significant (*r* = 0.37, *p* = 0.02) for the change in radial diffusity (Table 5). The relationship between ΔFA_genu_ and the enhancement of the sustained attention task may be difficult to interpret in smokers because the placebo session may not be measuring the cognitive baseline as the smokers could experience subtle withdrawal effects that may confounds their performance (Hendricks et al., 2006). Re-testing this effect in non-smokers may be critical to improve the credence to this observation.

**Table 5:** **Correlation coefficients for intersession difference in diffusivity values for the genu of corpus callosum and sustained attention hit rate and reaction time**.

	**Hit rate nicotine patch**	**Hit rate placebo patch**	**ΔHit rate**	**Reaction time nicotine patch**	**Reaction time placebo patch**	**ΔReaction time**
ΔL‖_genu_	0.01 (0.99)	−0.17 (0.30)	0.34 (0.05)^*^	0.28 (0.09)	−0.20 (0.23)	0.00 (1.0)
ΔL⊥_genu_	0.46 (0.004)^**^	0.37 (0.02)^*^	0.01 (0.99)	−0.23 (0.15)	−0.33 (0.20)	0.24 (0.15)

* *Nominally significant*.

** *Only the correlation with hit rate during the nicotine patch session was considered statistically significant after correction for N = 12 comparisons*.

Our observations have several limitations. The samples were heterogeneous in terms of diagnosis, medication exposure, demographics and duration and severity of smoking exposure, although our observations were based on within-subject design where these factors were unchanged. The discovery cohort consisted of schizophrenia patients and controls. However, there were no difference in the group's main effect and interaction. *Post-hoc* analysis in the discovery cohorts that was performed in normal controls replicated both the main effect of treatment and treatment × cotinine interaction (*p* = 0.02 and *p* = 0.01, respectively). Subjects in the discovery cohorts received 35 and 21 mg patches, based on their smoking habits, while all subjects in the replication cohort received 21 mg patch. The sample sizes were small to detect the more subtle pharmacological effects on DTI. The heterogeneous nature of the samples, especially the discovery sample, could have contributed to several observations unable to pass the corrections for multiple comparisons. We attempted to evaluate whether withdrawal symptoms, head motion and physiological measurements, such as the blood pressure, may have contributed to the main findings but none of these measures demonstrated to be significant predictors. However, subtle withdrawal symptoms, not detectable by the withdrawal scale, may still have produced measurable changes in sustained attention (Hendricks et al., 2006). Another potential limitation of this study is the use of cotinine level as predictors of nicotine intake from smoking because it is impacted by the treatment during the nicotine session. We believe this to be a minor limitation because long metabolic half life of cotinine makes it more sensitive to nicotine intake from smoking rather than the patch. This is demonstrated by high and consistent inter-session correlation in cotinine levels observed for both cohorts. Nonetheless, replication of this observation in non-smokers will be necessary to clarify this. Another limitation was that the DTI protocol only had 12 directions resulting in limited angular resolution compared to more modern high-angular resolution diffusion imaging (HARDI) protocols. This limitation was due to the use of an older MRI scanner that lacked the HARDI capability. We consider this to be a minor limitation since calculation of FA requires only six non-collinear directions. Use of lower angular direction protocols leads to lower signal-to-noise ratio in FA maps (Jones et al., 1999). We attempted to compensate for it by performing multiple averages (5 and 4 for discovery and replication cohorts, respectively). In conclusion, our observation remains novel and biologically based. The nicotine effect on FA can potentially be explained by pharmacological effects on axonal or glial or synaptic nAChRs that transiently alter ion concentration in or around the axonal pace to subtly influence WM integrity. Overall, this study should encourage and help with planning the future research to study the nicotine effects on brain and cognition. Specifically, evaluation of nicotine effects on WM integrity in non-smokers could remove confounds of past nicotine exposure and improve our understanding of biological mechanisms behinds the changes in FA values.

### Conflict of interest statement

The authors declare that the research was conducted in the absence of any commercial or financial relationships that could be construed as a potential conflict of interest.
